# Soil or Vermiculite-Applied Microencapsulated Peppermint Oil Effects on White Mustard Initial Growth and Performance

**DOI:** 10.3390/plants9040448

**Published:** 2020-04-03

**Authors:** Agnieszka Synowiec, Agnieszka Krajewska

**Affiliations:** 1 LeStudium Institute for Advanced Studies, 45000 Orléans, France; 2 Department of Agroecology and Crop Production, University of Agriculture in Krakow, 31-120 Kraków, Poland; 3 Institute of Natural Products and Cosmetics, Lodz University of Technology, 90-924 Łódź, Poland; agnieszka.krajewska@p.lodz.pl

**Keywords:** anthocyanins, botanical herbicide, dose-response test, maltodextrin

## Abstract

Microencapsulated peppermint (*Mentha x piperita* L.) essential oil (MPEO) is a prospective botanical herbicide. A hypothesis was formulated, that the type of growth medium (vermiculite or silty clay loam soil substrate) affects the phytotoxic potential of MPEO. A pot experiment in a randomized design assessed the effect of five doses of MPEO in a range of 0–108 g m^−2^ or 0–145 g m^−2^, mixed with vermiculite or with soil, respectively, on early growth of white mustard (*Sinapis alba* L. cv. Zlata), tested here as a model “weed” species. The morphologic analyses were supported by selected biochemical measurements. The two highest doses of microcapsules (from 73 to 145 g m^−2^) caused a significant decrease of plants’ height and biomass. An increase of anthocyanin content in the aboveground parts of mustard is supportive for the induction of defense mechanisms against MPEO-triggered stress in mustard leaves. In conclusion, MPEO appears as a promising bio-herbicide. However, we are aware that further studies on the mechanisms of action of MPEO in different weed species are necessary to test (i) whether or not the effect is consistent to be proficiently exploited for weed control in field and (ii) to deepen the biochemical and physiological reactions by the plants against MPEO treatments.

## 1. Introduction

Peppermint (*Mentha x piperita* L.), a natural hybrid of *Mentha aquatica* L. and *Mentha spicata* L., is cultivated broadly in the world. It is a valuable species due to production of essential oil (EO), applied in different industries [1,2]. In vitro studies showed, that peppermint EO displays also significant allelopathic and phytotoxic potential, by inhibiting germination and early growth of various weeds [3]. This potential is attributed to the main compounds of the peppermint EO-oxygenated monoterpenes, namely menthol and menthone constituting together 45%–80% of the oil [4]. According to Synowiec et al. [5], these compounds are mainly responsible for the allelopathic potential of EOs. However, some synergistic effects with the less representative compounds of the EO may also occur [6].

Essential oils of plant origin could play in future a significant role in the eco-friendly management of agricultural pests, including weeds [7,8]. As natural compounds, they are easily biodegradable in the environment at certain doses [9], by soil microbiota [10] and display different modes of action, in comparison to the synthetic pesticides [11,12]. On the other hand, their volatility is a significant constraint, as it leads to the fast evaporation of EOs before they can exert their allelopathic potential. Because of that, different methods, vide carriers, of EOs applications are sought to ensure their effectiveness for pre-emergence weed control. One of the promising venues is the application of EOs as solid preparations i.e., microcapsules or nanocapsules [13]. The solid carriers for essential oils tend to be a promising tool, as they enable a precise application and reduce losses of EOs [13]. Moreover, they can also extend the biologic action of EOs by a slow release to the soil or growth medium [3]. In the previous pot experiment, it was shown that peppermint EO encapsulated in maltodextrin with a small addition of gum Arabic and mixed with peat and sand (2:1 v/v), significantly inhibited initial growth of *Zea mays* L., *Echinochloa crus-galli* (L.) P. Beauv. and *Chenopodium album* L., at a dose equal to 20 g per m^−2^ [14].

In this experiment, we tested whether the peppermint oil microencapsulated in maltodextrin displays the phytotoxic potential against white mustard (*Sinapis alba* L., cv. Zlata) growing in vermiculate or in the top layer of an agronomic soil. White mustard has been chosen as a model “weed” species. It has been used for that purpose in the other experiments, as it is characterized by intensive growth and competition abilities against the other crops [15,16]. Vermiculate, a hydrous phyllosilicate mineral, has been chosen as a neutral growth medium, which is chemically and biologically inert [17] for comparison with the agricultural soil, common in the region of Chartres, France. A hypothesis was formulated, that the type of growth medium affects the phytotoxic potential of microencapsulated essential oil against white mustard, which is dose dependent.

## 2. Results

### 2.1. The Content and Chemical Composition of Peppermint Oil in the Microcapsules

The SEM photos revealed, that the analyzed microcapsules were composed of granules of different shapes and sizes, in a range of 5–70 μm (Figure S1 Supplementary).

Based on the hydrodistillation analysis, the average content of peppermint EO in the microcapsules was of 0.96 ml per 10 grams of microcapsules, equal to 9.6% of EO in the microcapsules (v/w). The chemical composition of the analyzed peppermint EO was in general typical for this oil, with the main compounds being menthol and menthone, constituting together ~80% of the oil. However, the oil contained higher amounts of menthol and lacked limonene oxide, as compared to the European Pharmacopoeia [18] (Table 1).

### 2.2. The Effect of MPEO in Vermiculate on Growth of Mustard

The growth of white mustard was unaffected by the presence of lower doses of MPEO (0–36 g m^−2^), mixed with the vermiculate. A visible drop in the number of emerging plants was observed only for the three highest doses of MPEO, namely 55–108 g m^−2^ (Table 2). Also, a significant reduction of their shoot length, by 57% and 85%, was registered only when the two highest doses of MPEO were incorporated. Elongation of roots was even less susceptible than shoots, as only the highest dose of MPEO (108 g m^−2^) caused a significant reduction of root length, by 68% as compared to the untreated control. On the other hand, the accumulation of biomass was more affected by the MPEO treatments and both fresh and dry mass of shoots and roots followed similar patterns. Significant reductions of fresh and dry biomass of shoots were registered for the three highest doses of MPEO, by ~40%, 57% and 90% for the doses of 55, 73 and 108 g m^−2^, respectively, as compared to control. The reductions of fresh mass of roots were of 75%, 81% and 84% and the reductions of dry mass of roots were of 46%, 70% and 88%, for the three highest doses of MPEO, respectively, in comparison to control (Table 2).

The analysis of ED50 and ED90, the doses responsible for respectively 50% and 90% reduction of a trait, confirmed, that the fresh mass of roots was most affected by the MPEO. Moreover, this analysis revealed also that shoot growth and biomass accumulation were inhibited by 50% by similar doses of MPEO, equal to ~71–73 g m^−2^ (Table 2).

### 2.3. The Effect of MPEO in Soil Substrate on Growth and Biochemical Characteristics of White Mustard

Similarly, as in the vermiculate experiment, the growth of shoots of white mustard in the silty clay loam soil substrate was affected significantly in the presence of the two highest doses of the MPEO, by 72% and 93% as compared to the non-treated control. Fresh and dry mass of shoots were significantly reduced only by the two highest doses, by 83%–88% and 87%–88% respectively, as compared to control. However, based on the ED50 value, accumulation of biomass in mustard shoots was more affected than shoot elongation under MPEO treatment (Table 3).

The fresh mass of roots was a parameter of a highest susceptibility to the presence of MPEO in the soil substrate, which was also confirmed by the calculated ED50 and ED90 values. A significant decrease of the root fresh mass was noted for all the treatments with MPEO in a range of 27%–91%, as compared to control. At the same time, a significant decrease of dry mass of roots was noted for the two highest doses of MPEO only, by 79% and 92%, as compared to control (Table 3).

For white mustard growing in the soil substrate, selected biochemical analyses were performed, to correlate the observed growth patterns with biochemical state of plants (Table 4). The higher doses of MPEO in the soil substrate, the higher the content of anthocyanins in the aboveground parts of white mustard. For the individual doses it was on average by about 61%; 100%; 75%; 115% and 126% higher, as compared to the untreated control, however significant differences were noted for the two highest doses of MPEO. Contrary, the content of total phenolic compounds was similar, regardless of the dose of MPEO; and for the two highest doses it was insignificantly higher by about 20%, as compared to the untreated control (Table 4).

## 3. Discussion

The chemical composition of microencapsulated peppermint EO (MPEO) studied in this experiment was in general in accordance with the recommendations of the European Pharmacopoeia [18], with monoterpene alcohol—menthol and monoterpene aldehyde—menthone, being its main compounds. At the same time, two compounds of the EO had an atypical concentration. First, the analyzed EO lacked limonene oxide and secondly—it contained higher amounts of menthol, as those recommended by the European Pharmacopoeia [18]. These differences could result from the previous processing of the EO namely its microencapsulation and further its hydrodistillation.

The results revealed that the fresh mass of roots of mustard was most reduced in the presence of soil applied MPEO, regardless of the type of medium. It could be speculated, that both phytotoxic peppermint EO, but also its carrier—maltodextrin, could be a reason for that phenomena. As was shown in the other experiment, peppermint oil affects the growth of radicles more, as compared to the growth of shoots [19]. Maltodextrin is a highly processed polysaccharide that can be enzymatically derived from any starch, most commonly made from corn, rice, potato starch or wheat [20]. Majority of research relate to its effect on humans, as it is a popular additive to many food products, e.g., it has a high glycemic index, higher than for a white sugar [21]. Maltodextrin is fully dissolved in water. It can by hypothesized that its addition to the soil medium could impair the sorption of water by the mustard roots, by potentially leading to an osmotic stress. That could be a reason of a lower fresh biomass of roots, which were poorly hydrated. This could potentially reduce the amount of water transported to the shoots, however, regardless of the type of growth medium used in this experiment, the growth of mustard cv. ‘Zlata’ was unaffected by the presence of lower doses of MPEO, up to 55 and 73 g m^−2^ for vermiculate and soil, respectively. Only the highest doses of MPEO, namely 73 and 108 g m^−2^ in the vermiculate experiment and 108 and 145 g m^−2^ in the soil experiment affected significantly both growth and biomass accumulation of mustard’s shoot. Similarly, in the field experiment carried out on brown podzolic soil, the inhibitory effect of maltodextrin (in doses of 50 and 100 g m^−2^) on the dry mass of dicotyledonous and monocotyledonous weeds was observed. The authors correlated this effect with possible maltodextrin-caused changes in the activity of soil microbiota, especially a decrease in the number of colonies of mesophilic bacteria, fungi and actinomycetes [10].

It was also shown that mustard growing in the presence of vermiculate was higher and had a more hydrated tissues but also it accumulated less biomass, as compared to the mustard growing in the silty clay loam soil substrate. Actually, mustard was more tolerant to the presence of MPEO in the soil than in the vermiculite. Vermiculite has a good capacity to store water in a way that is easily available for plants [16]. That property could stimulate a faster growth of mustard. On the other hand, silty clay loam soil, used in this experiment, contained high amounts of nutrients, which promoted the buildup of plants’ biomass. Similar relationships of biomass accumulation and plants’ length of cucumber seedlings in the presence of vermiculate as compared to the other types of growth media were noted by Kleifeld and Chet [22].

At the same time, the question raises about the mechanisms of tolerance of mustard to the soil-applied peppermint oil. The biochemical tests performed in this experiment showed, that there was a significant increase in the content of total anthocyanins in the mustard’s tissues in the presence of the two highest doses of the MPEO. This phenomenon is supportive for the induction of stress-alleviating mechanisms in mustard due to phytotoxic action of the MPEO. As was showed in the other experiments, anthocyanins’ production increases in the presence of different types of stresses [23,24]. Indeed, the presence of these compounds can ameliorate plant performances reducing the excess lighting striking chloroplast when the photosynthetic apparatus is under sub-optimal conditions (e.g., MPEO-triggered biochemical and physiological effects) as observed in other experiments dealing with environmental stresses [25]. On the other hand, the content of phenolic compounds, which are also responsible for the allelopathic stress [26], was similar for both control and the MPEO-treated plants.

Perhaps, a unique chemical composition of mustard seeds could also be a reason for its higher tolerance to the MPEO. Mustard belongs to oilseed crops; its seeds contain approx. 28% of fatty acids, but also 0.1%–1.1% of essential oil [27]. As was showed in the other laboratory experiment, seedlings of another oilseed species—*Brassica napus* were able to germinate in the presence of different essential oils more, as compared to the other non-oilseed species [5]. Indeed, this phenomenon requires more in-depth studies.

## 4. Conclusions

In summary, the model “weed”—white mustard cv. Zlata showed a tolerance to the presence of maltodextrin microencapsulated peppermint oil (MPEO) in the vermiculate and silty clay loam soil substrate, up to 55 and 73 g m^−2^, respectively. Only the two highest doses of microcapsules, namely 73 and 108 g m^−2^ in the vermiculate experiment and 108 and 145 g m^−2^ in the soil experiment, caused a significant decrease of plants height and biomass concentration in shoots and roots. The growth patterns were followed by the biochemical changes in the mustard’s tissues, namely a significant increase of anthocyanin content by about 61%–126%. The increase of anthocyanins points to the induction of defense mechanisms in mustard against the MPEO-triggered stress. MPEO treatment appears as a promising bio-herbicide which can be proficiently exploited for weed control in field. However, further studies on different weeds are necessary to consistently evaluate the target of action of MPEO in plant biochemical and/or physiological pattern(s).

## 5. Materials and Methods

### 5.1. Characterization and Chemical Analysis of Microcapsules

Microencapsulated peppermint essential oil (MPEO), obtained by the method of a dry spraying, was purchased in 2017 from the producer (Hoffmann Aroma, Zamysłowo, Poland). The carrier for the EO was maltodextrin with a small addition (4.5%) of gum Arabic E414.

The content of peppermint EO in the microcapsules was measured three times by the hydrodistillation method (10 g of microcapsules and 100 mL of water) for 2 h, using a Clevenger-type apparatus. The volume of the separated EO was multiplied by the specific density of the microcapsules, which was determined by the pycnometer method. Essential oil was analyzed by gas chromatography coupled with mass spectrometry (GC-FID-MS), using a Trace GC Ultra gas chromatograph coupled with DSQ II mass spectrometer (Thermo Electron Corporation). The operating conditions were as follows: non-polar capillary column Rtx-1ms (60 m × 0.25 mm, 0.25 m film thickness), programmed temperature: 50 (3 min)–300 °C, 4 °C/min, injector (SSL) temperature 280 °C, detector (FID) temperature 300 °C, transfer line temperature 250 °C, carrier gas-helium, flow with constant pressure 200 kPa, split ratio 1:20. The mass spectrometer parameters: ion source temperature 200 °C, ionization energy 70 eV (EI), scan mode: full scan, mass range 33–420. The percentages of constituents were computed from the GC peak area without using a correction factor.

Identification of the components was based on a comparison of their mass spectra and linear retention indices (RI, non-polar column), determined with reference to a series of n-alkanes C8-C24, by comparing with those in Adams [28] as well as in computer libraries: NIST 2011 and MassFinder 4.1.

The photos of MPEO were performed with a LEO 1430 VP, a standard scanning electron microscope (SEM) with secondary electron detector, at room temperature.

### 5.2. Pot Experiments

Two dose-response pot experiments were set up in the Spring–Summer 2019 in the greenhouse of the Pôle Universitaire in Chartres (France), without regulated light and temperature. Bottom of each pot (1 L vol.) was lined with a layer of a standard filter paper, to prevent leaking. White mustard (*Sinapis alba* L.) cv. Zlata (purchased from Caillard, www.graines-caillard.com) was the experimental model “weed”.

In the first experiment, a mixture of the medium- and small-sized vermiculate (GRANUTEC^®^ E, www.cmmp.fr) in a proportion 1:1 (v/v) was used as a soil-substrate. Next, different doses of MPEO were added to the pots: 0 (control), 0.1; 0.2; 0.44; 0.66; 0.88 and 1.31 g per pot (equal to 8; 16; 36; 55; 73 and 108 g m^−2^) and mixed with the upper part of vermiculite (up to 3-cm deep). On the same day, three seeds of white mustard were seeded in each of the pots (at a depth of a ~1 cm) and after emergence they were thinned to two per pot.

Based on the growth parameters of mustard in the first experiment, the next experiment with soil as a substrate, was set up. In this experiment, a top layer (~20-cm deep) of a clay-loamy soil was collected from the organic field during Autumn 2018 at La Saussaye farm near Chartres, France. The soil was kept outside in a box until Spring and then air-dried and sieved through a 2-cm mesh, to remove all the impurifications. The granulometric and chemical analyses of soil were performed in the Laboratoire d’Analyses Chambre d’Agriculture Loiret in Orleans, France. The soil based on its texture was classified as a silty clay loam [29]. It contained: C_org_—12.7 g kg^−1^; P_2_O_5_—86 mg kg^−1^; K_2_O—270 mg kg^−1^; N_total_—1.38 g kg^−1^; pH—7.93. Different doses of MPEO were added to pots containing the soil: 0 (control), 0.44; 0.66; 0.88, 1.31 and 1.76 g per pot (equal to 36; 55; 73; 108; 145 g m^−2^) and mixed with the upper part (~3-cm deep) of the soil-substrate. Analogically, as for the first experiment, on the same day three seeds of white mustard were seeded in each of the pots and after emergence thinned to two per pot.

Plants in the both experiments were watered with a tap water every 2–3 days. No fertilization was applied, to prevent a potential hindering and/or interaction between fertilized nutrients and the microcapsules.

### 5.3. Plant Measurements

Both experiments were terminated when the white mustard plants reached the growth stage of two pairs of true leaves (BBCH 14), which took ~8 weeks, as the emergence of plants was registered ~10–12 days after seeding. The plants were removed from the pots and their roots were carefully washed under tap water. The measured parameters of fresh plants included: length of shoots, length of roots (for the vermiculate experiment only) and fresh mass of shoots and roots. Also, a dry mass of shoots and roots of mustard was measured, after drying plants in 105 °C for 24 h.

From the experiment with the soil substrate, three plants were used for the biochemical analyses. Methanolic extracts (50%) of lyophilized aboveground parts of mustard were prepared using ultrasound-assisted extraction for 60 min (Prolab Instruments GmbH, Kanton Reinach, Switzerland). The biochemical analyses were performed in two technical repetitions and included photocolorimetric analyses (BioTek Instruments Inc., Winooski, VT, USA), using 96-micro well plates. The Folin–Ciocalteu assay of total phenolic content with absorbance readings at 630 nm was taken after incubation and expressed in gallic acid as a reference phenolic [30]; total anthocyanins were measured using the pH differential method at 450 nm [31].

### 5.4. Statistical Analyses

The experiment with vermiculate was set up in two series, which begun on 11 April and on 22 April 2019, respectively; whereas the experiment with soil was set up on 27 May 2019. Both pot experiments were set up in a totally randomized design, with four replications (pots) for the experiment with vermiculate and three replications (pots) for the experiment with the soil substrate. Each pot contained two plants of white mustard. Since no significant differences between the two series of the experiment with vermiculate were found, the data were pooled. The statistical analysis was based on ANOVA (software Statistica PL version 13.0, StatSoft). To meet the requirements of ANOVA namely normality of the distribution, the data for shoot and root length were square root transformed. Means were compared using Tukey HSD test at *p* < 0.05. The ED50 and ED90 values were calculated using the ‘drc’ package in the RStudio (ver. 1.2.5033) software [32].

## Figures and Tables

**Table 1:** Chemical composition of peppermint oil encapsulated in the maltodextrin microcapsules.

Compound	RI Lit ^1^	RI Exp ^2^	RT (min) ^3^	Av. Content (%)	EP ^4^ (%)
α-Pinene	934	930	11.79	0.37	
Sabinene	970		13.25	0.10	
β-Pinene	974	969	13.34	0.60	
1,8-Cineol	1024	1017	15.38	4.17	3.5–14.0
Limonene	1025	1021	15.48	0.63	
Limonene oxide				-	0.1–5.0
Menthone	1139	1134	20.14	20.63	14.0–32.0
Isomenthone	1046	1142	20.40	3.73	1.5–10.0
Menthofuran	1050			t ^5^	0.1–0.9
Neomenthol	1155	1150	20.71	2.53	
Menthol	1163	1166	21.40	60.10	30.0–55.0
Neoisomenthol	1171	1170	21.52	0.43	
Isomenthol	1176	1176	21.73	0.17	
Pulegone	1218	1215	23.34	0.20	< 4.0
Piperitone	1232	1233	23.81	0.20	
Menthyl acetate	1280	1275	25.56	4.00	
Bicycloelemene	1338	1328	27.51	0.10	
β-Bourbonene	1386	1378	29.27	0.10	
(E)-β-Caryophyllene	1421	1410	30.40	0.40	
ε-Muurolene	1455	1445	31.48	0.10	
Caryophyllene oxide	1573	1568	35.40	0.93	

^1^ RI lit—standard retention index; ^2^ RI exp.—experimental retention index; ^3^ RT—retention time; ^4^ EP—European Pharmacopoeia [18]; ^5^ t—trace < 0.05%.

**Table 2:** Length of white mustard cv. ‘Zlata’ shoots and roots and their biomass at the growth stage of two pairs of true leaves (BBCH 14) after 8 weeks of growth in vermiculite amended with different doses of microencapsulated peppermint essential oil (MPEO with 9.6% (v/w) of peppermint EO content).

MPEO Dose (g m^−2^)	Percent of Emerged Plants	Length (cm) ^1^		Fresh Mass (g)		Dry Mass (g)	
		Shoot	Root	Shoot	Root	Shoot	Root
0	100	22.9 ± 1.09a	25.4 ± 0.27a	0.40 ± 0.02a	0.16 ± 0.015a	0.053 ± 0.002a	0.026 ± 0.002a
8	100	21.2 ± 1.83a	26.0 ± 2.26a	0.32 ± 0.03a	0.05 ± 0.008b	0.045 ± 0.003a	0.020 ± 0.001a
16	100	15.1 ± 0.92a	24.6 ± 2.37a	0.24 ± 0.03b	0.05 ± 0.015b	0.033 ± 0.003b	0.017 ± 0.003a
36	100	16.0 ± 1.76a	34.1 ± 1.61a	0.32 ± 0.03a	0.11 ± 0.034a	0.040 ± 0.004a	0.020 ± 0.002a
55	88	15.2 ± 2.45a	30.6 ± 5.17a	0.24 ± 0.04b	0.04 ± 0.016b	0.032 ± 0.005b	0.014 ± 0.003b
73	63	9.8 ± 3.03b	17.8 ± 5.34a	0.17 ± 0.05b	0.03 ± 0.012b	0.023 ± 0.007b	0.008 ± 0.003b
108	25	3.5 ± 2.45b	8.1 ± 5.30b	0.04 ± 0.03b	0.01 ± 0.004b	0.007 ± 0.005b	0.003 ± 0.002b
**ED50 ^2^**	**83.2 ± 0.46**	**71.7 ± 9.75**	**87.5 ± 9.11**	**73.0 ± 62.9**	**3.48 ± 5.97**	**72.1 ± 6.82**	**64.4 ± 5.85**
**ED90 ^2^**	**136.5 ± 1.67**	**157.8 ± 76.8**	**129.6 ± 23.9**	**119.1 ± 23.2**	**142.7 ± 170**	**131.0 ± 31.0**	**114.3 ± 26.3**

Values are means (±SE) that were pooled for both series with four replications of the pot experiment. Different letters in columns denote significant differences between control (0) and the treatments, according to Tukey HSD test at *p* < 0.05. ^1^ ANOVA was performed on the square root-transformed data. The table contains rough values. ^2^ ED50 and ED90 stand for effective doses causing 50% and 90% reduction of a particular trait (±SE).

**Table 3:** Length of white mustard cv. ‘Zlata’ shoots and roots and their biomass at the growth stage of two pairs of true leaves (BBCH 14) after 8 weeks of growth in a silty clay loam soil substrate amended with different doses of microencapsulated peppermint essential oil (MPEO with 9.6% (v/w) of peppermint EO content).

MPEO Dose (g m^−2^)	Percent of Emerged Plants	Shoot Length (cm) ^1^	Fresh Mass (g)		Dry Mass (g)	
			Shoot	Root	Shoot	Root
0—control	100	13.4 ± 0.64a	0.87 ± 0.082a	0.11 ± 0.018a	0.08 ± 0.009a	0.024 ± 0.003a
36	100	15.1 ± 1.07a	0.67 ± 0.094a	0.08 ± 0.010b	0.07 ± 0.011a	0.019 ± 0.002a
55	100	13.2 ± 1.05a	0.43 ± 0.067b	0.05 ± 0.005b	0.04 ± 0.008b	0.015 ± 0.002a
73	100	15.0 ± 0.89a	0.53 ± 0.019a	0.04 ± 0.003b	0.05 ± 0.003a	0.016 ± 0.002a
108	30	3.8 ± 2.46b	0.15 ± 0.097b	0.02 ± 0.010b	0.01 ± 0.008b	0.005 ± 0.003b
145	17	1.0 ± 1.00b	0.11 ± 0.106b	0.01 ± 0.008b	0.01 ± 0.007b	0.002 ± 0.002b
**ED50 ^2^**	**99.3 ± 1.38**	**102.6 ± 11.5**	**65.7 ± 10.1**	**56.0 ± 7.09**	**72.4 ± 10.1**	**76.2 ± 11.4**
**ED90 ^2^**	**130.1 ± 4.33**	**114.8 ± 15.9**	**177.6 ± 44.1**	**136.8 ± 28.4**	**155.2 ± 32.1**	**158.6 ± 34.0**

Values are means ±SE of three replications. Different letters in columns denote significant differences between control and the treatments, according to Tukey HSD test at *p* < 0.05. ^1^ ANOVA was performed on the square root-transformed data. The table contains rough values. ^2^ ED50 and ED90 stand for effective doses causing 50% and 90% reduction of a particular trait (± SE).

**Table 4:** Biochemical characteristic of aboveground parts of white mustard cv. ‘Zlata’ at the growth stage of two pairs of leaves (BBCH 14), growing in the silty clay loam soil substrate and in the presence of different doses of microencapsulated peppermint essential oil (MPEO with 9.6% (v/w) of peppermint EO content).

MPEO Dose (g m^−2^)	Total Anthocyanins (µg g^−1^ DW)	Total Phenolics (µg g^−1^ DW Gallic Acid Eq.)
0—control	22.9 ± 2.00a	570 ± 36.2a
36	36.9 ± 3.89ab	581 ± 31.3ab
55	46.2 ± 4.78b	707 ± 38.5b
73	40.0 ± 2.02ab	599 ± 34.0ab
108	49.2 ± 6.81b	682 ± 4.6ab
145	51.8 ± 4.87b	642 ± 25.8ab

Table contains mean values ±SE. Different letters in columns denote significant differences between control and the treatments, according to Tukey HSD test at *p* < 0.05.

## Supplementary Materials

The following are available online at https://www.mdpi.com/2223-7747/9/4/448/s1, Figure S1: SEM photo of the microencapsulated peppermint essential oi. Photo credentials: Dr. J.P. Blondeau.
