# Supplementary material for: Soil or Vermiculite-Applied Microencapsulated Peppermint Oil Effects on White Mustard Initial Growth and Performance
Source: Plants (Basel). 2020 Apr 3;9(4):448. doi: 10.3390/plants9040448 (PMC7238143; doi:10.3390/plants9040448)
Supplement: Supplementary file 1 [file plants-09-00448-s001.pdf]

plants

MDPI

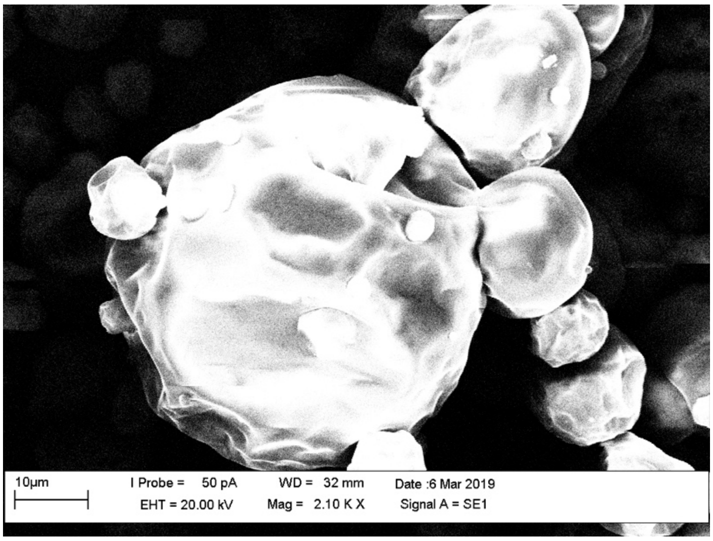

Figure S1. SEM photo of the microencapsulated peppermint essential oi. Photo credentials: Dr. J.P. Blondeau.
